# Prevalence of spontaneous bacterial peritonitis in liver cirrhosis with ascites

**DOI:** 10.11604/pamj.2013.15.128.2702

**Published:** 2013-08-09

**Authors:** Ajayi Akande Oladimeji, Adegun Patrick Temi, Ajayi Ebenezer Adekunle, Raimi Hassan Taiwo, Dada Samuel Ayokunle

**Affiliations:** 1 Department of Medicine, Ekiti State University Teaching Hospital, P.M.B 5355, Ado Ekiti, Nigeria; 2 Department of Surgery, Ekiti State University Teaching Hospital, P.M.B 5355, Ado Ekiti, Nigeria

**Keywords:** Spontaneous bacterial peritonitis, abdominal paracentesis, liver cirrhosis, ascites

## Abstract

**Introduction:**

Spontaneous bacterial peritonitis (SBP) is a common bacterial infection in patients with cirrhosis and ascites requiring prompt recognition and treatment. The aim of this study was to determine the prevalence, and characteristics of SBP among in-patients with cirrhosis and ascites seen at our facility.

**Methods:**

Thirty one patients with liver cirrhosis and ascites who were admitted into the Medical ward of the Ekiti State University Teaching Hospital (EKSUTH), Ado-Ekiti, Nigeria from August 2009 to July 2010 were retrospectively studied. All the patients had abdominal paracentesis done within 48 hours of admission under aseptic condition and the data obtained were analyzed.

**Results:**

The mean age of the studied population was 62±9 years (age range 43-78 years). Of the 21 that developed SPB, culture positive SBP was present in 66.7% (14/21) while CNNA was found in 33.3% (7/21). The prevalence of MNB was 26% (8/31) in this study. Of those with SBP, 93% had monomicrobial infection with aerobic Gram negative bacilli being responsible in 66.7% of the cases with *E.coli* (70%) being the predominant organism followed by Klebsiella species. Gram positive organisms accounted for 33.3% with Streptococcal species (60%) being the predominant organism followed by *Staphylococcus aureus* (40%). Patients with SBP had significantly lower platelet count when compared with those without SBP, p < 0.05. Also, international normalization ratio (INR) was significantly higher in those patients with SBP compared with those without SBP, p < 0.05. The poor prognostic indicators found in this study were; low ascitic protein, hepatic encephalopathy, coagulopathy, renal dysfunction (creatinine >2mg/dl) and leukocytosis (p < 0.05).

**Conclusion:**

It is therefore imperative to do diagnostic abdominal paracentesis for cell count and culture in any patient with onset of ascites or cirrhotic patients with ascites and suggestive symptoms compatible or suggestive of SBP.

## Introduction

Spontaneous bacterial peritonitis (SBP) is a very common bacterial infection in patients with cirrhosis and ascites requiring prompt recognition and treatment [1–3]. It was first described by Conn and Fessel in 1971 as a syndrome of infected ascitic fluid in patients with hepatic cirrhosis, which they named SBP [4]. SBP is by definition an infection of previously sterile ascitic fluid, without any apparent intra-abdominal source of infection [5]. The infecting organisms are usually those found among the normal intestinal flora. When first described, its mortality exceeded 90% but it has been reduced to approximately 20% with early diagnosis and treatment [6–7].

All patients with cirrhosis and ascites are at risk of SBP and the prevalence of SBP in outpatients is 1.5-3.5% and about 10%-30% in hospitalized patients [8–9]. Half of the episodes of SBP are present at the time of hospital admission while the rest are acquired during hospitalization [1]. In-hospital mortality for the first episode of SBP ranges from 10% to 50%, depending on various risk factors [10–11]. One-year mortality after a first episode of SBP has been reported to be 31% and 93% [12].

The pathophysiology of SBP is not completely understood. Translocation of bacteria and endotoxins from the gastrointestinal tract to peritoneal fluid is believed to be a key mechanism behind the development of SBP, and is facilitated by impaired defensive mechanisms in cirrhotic patients [13–17]. Proteins of the complement cascade have lower levels in cirrhotic patients and the opsonic and phagocytic properties of neutrophils are decreased in patients with cirrhosis. Bacteremia from the urine or the respiratory tract can also lead to infection of the ascitic fluid. SBP may also be iatrogenic [18], such as after endoscopic treatment of esophageal or gastric varices.

Patients with SBP may have one of the following [1, 8]: (1) local symptoms and/or signs of peritonitis: abdominal pain, abdominal tenderness, vomiting, diarrhea, ileus; (2) signs of systemic inflammation: hyper or hypothermia, chills, altered white blood cell count, tachycardia, and/or tachypnea; (3) worsening of liver function; (4) hepatic encephalopathy; (5) shock; (6) renal failure; and (7) gastrointestinal bleeding. However, it is important to point out that SBP may be asymptomatic in about 10-32% of cases, particularly in outpatients [8, 19–21]. Nigerian data is very scanty regarding the prevalence of SBP in patients with liver cirrhosis and ascites. The aim of this study was to determine the prevalence and characteristics of SBP among in-patients with cirrhosis and ascites seen at our facility and to compare the results with studies elsewhere.

## Methods

Thirty one patients with liver cirrhosis and ascites who were admitted into the Medical ward of the Ekiti State University Teaching Hospital (EKSUTH), Ado-Ekiti, Nigeria from August 2009 to July 2010 were retrospectively studied. The diagnosis of liver cirrhosis was established on the basis of clinical evaluation, biochemical parameters, abdominal ultrasounds and viral markers. All the patients had abdominal paracentesis done within 48 hours of admission under aseptic condition. Ascitic fluid analysis was done that included absolute neutrophils count, culture sensitivity, ascitic albumin and cytology.

SBP was diagnosed using standard criteria, namely, an absolute neutrophil count of >250 cells/mm^3^ as determined by microscopy, that is neutrocytic ascites, in the absence of an intra-abdominal source of infection [1, 22]. If ascitic fluid cultures were positive and the neutrophil count was >250 cells/mm^3^, such patients were diagnosed as having culture- positive neutrocytic ascites or SBP. If ascitic fluid cultures were negative in the presence of neutrocytic ascites, these patients were characterized as having culture-negative neutrocytic ascites (CNNA). Patients with positive cultures on ascitic fluid but without neutrocytic ascites were classified as having monobacterial bacterascites (MNB).

An ethical clearance for this study was obtained from the EKSUTH Ethical and Research Committee. Data obtained were analyzed using the statistical package for social sciences (SPSS, version 15) statistical software.

## Results

The mean age of the studied population was 62±9 years (age range 43-78 years). Seventeen (54.8%) were males while fourteen (45.2%) were females giving a male to female ratio of 1.2:1. Fifteen (48.4%) were in the 41-60 age group while the remaining sixteen (51.6%) were in the 61-80 age group (Table 1). SBP developed in 21 (67.7%) of the patients. Of the 21 that developed SPB, culture positive SBP was present in 66.7% (14/21) while CNNA was found in 33.3% (7/21). The prevalence of MNB was 26% (8/31) in this study (Table 2).

**Table 1:** Age and gender distribution of the study population

Age Group	Male	Female	Total
41–60	9	6	15
61–80	8	8	16
**Total**	17	14	31

**Table 2:** Showing ascitic culture and neutrophil counts

Culture	Ascitic Neutrophils		Total
	<250/mm^3^	>250mm^3^	
Positive	8	14	22
Negative	2	7	9
Total	10	21	31

Of those with SBP, 93% had monomicrobial infection with aerobic Gram negative bacilli being responsible in 66.7% of the cases with *E. coli* (70%) being the predominant organism followed by Klebsiella species. Gram positive organisms accounted for 33.3% with Streptococcal species (60%) being the predominant organism followed by *Staphylococcus aureus* (40%) (Table 3).

**Table 3:** Showing bacteriology and culture of ascitic fluid

Bacteriology	Culture Positive	Culture Negative	Total
*Klebsiella spp*	4	0	4
*E.coli*	11	0	11
*Strept spp*	4	0	4
*Staph aureus*	3	0	3
Mixed growth	0	1	1
No growth	0	8	8
**Total**	22	9	31

**Figure 1:**
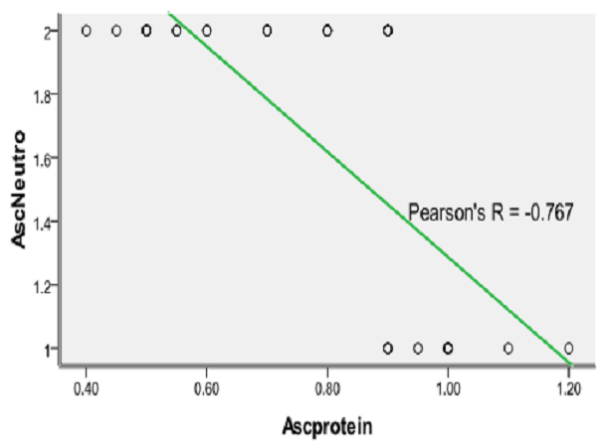
Showing the relationship between ascitic neutrophils and protein

There was no gender difference in the occurrence of SBP. All the patients with SBP had ascitic fluid protein less than 1gm% (Figure 1). Majority (80%) of the patients with SBP were in Child's grade C, while the remaining 20% were in Child's grade B. Patients with SBP had significantly lower platelet count when compared with those without SBP, p < 0.05. Also, international normalization ratio (INR) was significantly higher in those patients with SBP compared with those without SBP, p < 0.05. The poor prognostic indicators found in this study were; low ascitic protein, hepatic encephalopathy, coagulopathy, renal dysfunction (creatinine >2mg/dl) and leukocytosis (all having p < 0.05).

## Discussion

Spontaneous bacterial peritonitis (SBP) is a potentially life-threatening complication in patients with cirrhosis and has typically been described in hospitalized patients. The prevalence of SBP in hospitalized patients with cirrhosis and ascites is between 10% and 30% [23–24]. The prevalence of SPB in our study was found to be 67.7%. This was high compared to 10-30% found by most studies from the west; Tandon P et al [7], Rimola A et al [8], Almdal TP et al [23] and Pinzello G et al [24]. This may be due the fact that majority of our patients presented in Child's grade C. The prevalence of CNNA (a variant of SPB) in this study was 33.3% and this was similar to that reported by Runyon et al [25].

The prevalence of MNB in this study was 26%, higher than the 11% prevalence reported by Chu CM et al [26]. Again this might be due to the late presentation of the patients to our health facility.

In approximately 50-60% of cases, the organism responsible is isolated in ascitic fluid or in blood cultures, the remaining are variants of SBP [27]. More than 92% cases of SBP are monomicrobial, with aerobic Gram- negative bacilli; being responsible for more than two- third of all cases with *Escherichial coli* being the most common followed by Klebsiella species [28]. Almost 25% were caused by Gram-positive organisms with Streptococcal species being the most common followed by *Staphylococcus aureus*. Anaerobe causes nearly 1% of SBP and monobacterial ascites [29]. Anaerobes were not found in our study as a cause of SBP.

About 93% of the patients in this study had monomicrobial infection with aerobic Gram negative bacilli being responsible in 66.7% of the cases and this was similar to that reported by Gills AS et al [30]. E.coli was the predominant organism (70%), followed by Klebsiella species. E.coli was also the predominant organism found in the studies of Gills et al [30]. In the study conducted in Khyber Teaching Hospital, Peshawar 2003, showed E. coli was isolated in 58.13%, *Streptococcus pneumoniae* in 18.60%, Staphylococcus aureus in 9.13%, Klebsiella in 9.13% and Acinectobacter in 4.63% [31]. Gram positive organisms accounted for 33.3% with Streptococcal species (60%) being the predominant organism followed by *Staphylococcus aureus* (40%). The clinical presentations found in this study were; fever (70%), abdominal pain (56%), encephalopathy (38%) and jaundice (23%). This was consistent with the findings of Runyon BA et al [28].

The poor prognostic indicators found in this study were; low ascitic protein, hepatic encephalopathy, coagulopathy, renal dysfunction (creatinine <2mg/dl) and leukocytosis (all having p < 0.05). Majority of those that presented in Child's grade C succumbed to the overwhelming infection, again stressing the need for early presentation and high index of suspicion among the caring physicians.

## Conclusion

In view of the high prevalence and mortality rate of SBP in our study population, it is imperative that awareness campaign be vigourously pursued as regards early presentation to reduce the morbidity and mortality in this group of patients. It is equally recommended that diagnostic abdominal paracentesis for cell count and culture in any patient with onset of ascites or cirrhotic patients with ascites and suggestive symptoms compatible or suggestive of SBP be carried out.
